# Kisspeptin-mediated improvement of sensitivity to BRAF inhibitors in vemurafenib-resistant melanoma cells

**DOI:** 10.3389/fonc.2023.1182853

**Published:** 2023-09-18

**Authors:** Carlotta Guzzetti, Cristina Corno, Elisabetta Vergani, Luca Mirra, Emilio Ciusani, Monica Rodolfo, Paola Perego, Giovanni L. Beretta

**Affiliations:** ^1^ Molecular Pharmacology Unit, Department of Experimental Oncology, Fondazione IRCCS Istituto Nazionale dei Tumori Milan, Milan, Italy; ^2^ Unit of Immunotherapy of Human Tumors, Department of Experimental Oncology, Fondazione IRCCS Istituto Nazionale dei Tumori Milan, Milan, Italy; ^3^ Laboratory of Clinical Pathology and Medical Genetics, Istituto Neurologico Fondazione C. Besta, Milan, Italy

**Keywords:** drug resistance, melanoma, metastasis, kisspeptins, BRAF, vemurafenib

## Abstract

Metastatic dissemination is still one of the major causes of death of melanoma’s patients. KiSS1 is a metastasis suppressor originally identified in melanoma cells, known to play an important physiological role in mammals’ development and puberty. It has been previously shown that expression of KiSS1 could be increased in lung cancer cells using epigenetic agents, and that KiSS1 could have a pro-apoptotic action in combination with cisplatin. Thus, the aim of the present study was to examine in human melanoma vemurafenib sensitive- and -resistant BRAF mutant cells characterized by different mutational profiles and KiSS1, KiSS1 receptor and KiSS1 drug-induced release, if peptides derived from KiSS1 cleavage, i.e., kisspeptin 54, could increase the sensitivity to vemurafenib of human melanoma, using cellular, molecular and biochemical approaches. We found that kisspeptin 54 increases vemurafenib pro-apoptotic activity in a statistically significant manner, also in drug resistant cellular models. The efficacy of the combination appears to reflect the intrinsic susceptibility of each cell line to PLX4032-induced apoptosis, together with the different mutational profile as well as perturbation of proteins regulating the apoptotic pathway, The results presented here highlight the possibility to exploit KiSS1 to modulate the apoptotic response to therapeutically relevant agents, suggesting a multitasking function of this metastasis suppressor.

## Introduction

The metastasis suppressor KiSS1, originally identified in melanoma, plays a role in tumor cells (1–3), besides contributing to the neuroendocrine control of reproduction (4). The products of the metastasis suppressor gene KiSS1, namely KiSS1-derived peptides (i.e., kisspeptins), are secreted and interact with the KiSS1R/GPR54 receptor (5, 6). The proteolytic cleavage of the 145-aa polypeptide KiSS1 produces the 54-aa peptide, kisspeptin-54 (KP54)/metastin, which is further cleaved into shorter peptides, including kisspeptin-10, KP10; kisspeptin-13, KP13; kisspeptin-14, KP14 (6, 7). These cleaved peptides are secreted and retain their biological activity. KiSS1 has been reported to down-regulate the matrix metalloproteinases (2) and in such a way to inhibit metastasis of cancer cells (2). Although the role of KiSS1 in cancer is not completely clarified, an involvement in controlling metastasis dissemination and response to cisplatin (cDDP) has been proposed (8–10). In melanoma, the metastasis suppression properties of KiSS1 have been reported to counteract metastatic colonization and to control the dormancy of disseminated cells following secretion (5, 11). The loss of KiSS1 in tumor progression/metastases has been associated with other cancer types in addition to melanoma (12). A link between KiSS1 expression and epigenetic mechanisms (e.g., histone acetylation, DNA methylation, microRNAs expression) has been suggested (13, 14). For instance, we have reported that the up-regulation of KiSS1 mRNA levels stimulated by the treatment with histone deacetylase (HDAC) inhibitors resulted in a reduction of the invasive ability of the cDDP-resistant cells (13). More recently, another study from our research team showed a peculiar modulation of KiSS1 levels in liquid biopsies of non-small cell lung cancer (NSCLC) patients, supporting the potential use of KiSS1 as a biomarker for this tumor. The study also highlights the role played by KiSS1-cleaved peptide KP54 in increasing the apoptosis induced by the treatment with cDDP, envisioning possible implications for the use of KP54 in antitumor therapy of this disease (10).

Melanoma is an aggressive disease, responsible for the majority of deaths for skin cancers (15). Though the amelioration of the medical intervention has declined patients mortality, metastatic disease still remains incurable (16). Due to the frequent activating mutation of the BRAF gene, the constitutive activation of the RAS-RAF-MEK-ERK signalling is very common in melanoma. The mutation BRAFV600E, which is found in 40% of melanoma patients, is mainly responsible for melanoma aggressiveness. Patients suffering from metastatic disease have benefited from the introduction in clinical practice of the BRAFV600E kinase inhibitor PLX4032 (vemurafenib) (16, 17). However, these patients develop resistance to vemurafenib within 6–9 months (18) because of the reactivation of the MAPK pathway. In BRAF inhibitor-resistant patients, positive results have been reported by the co-treatment with BRAF and MEK inhibitors. Though the amelioration of the medical intervention by kinase inhibitors and by immunotherapy has declined patients mortality, the advanced metastatic disease still remains incurable (16). Unfortunately, the development of resistance toward the drug combination has limited the achievement of persistent cures (19). In this context, the development of new drugs, as well as innovative therapeutic strategies, is urgent. To face this issue, intensive efforts have been made to better understand the molecular bases of drug resistance in melanoma. Several studies assessing the genomic correlates of resistance to BRAF/MEK inhibitors in patients showed that the development of resistance is a complex process that may display a wide intra-patient and intra-tumoral heterogeneity of underlying mechanisms (20). Pre-clinical studies carried out in cell lines with primary or acquired resistance as model systems have enabled the dissection of molecular mechanisms that act by sustaining MAPK signaling or parallel signaling networks despite BRAF inhibition. *In vitro* studies identified different epigenetic, metabolic, and phenotypic reprogramming events associated to resistance, contributing to the definition of the heterogeneous alterations associated with the reactivation of MAPK signaling (21). In addition, these model systems represent a tool to develop novel drug combinations to improve precision medicine strategies.

Here, we gain further inside to the role played by KiSS1 in modulating the apoptotic response of melanoma cells to antitumor drug exposure and envision a possible combination of vemurafenib with kisspeptins for improving the response to chemotherapy treatment.

## Methods

### Cell lines and cell sensitivity to antitumor agents

The melanoma cell lines LM16 and LM36, were obtained at the Fondazione IRCCS, Istituto Nazionale dei Tumori of Milan from fresh surgical specimens of a nodal and a cutaneous metastases (22). The corresponding PLX4032-resistant sublines, LM16R and LM36R, were generated by treating the parental counterparts with PLX4032 (3.2 μM) for 96 hours, allowing the few surviving cells to re-grow, and repeating treatment for up to 11 times, until the setting of drug resistance. Their genetic molecular and phenotypic characterization has been reported in previous studies (23–27). In particular, all cell lines exhibited the V600E BRAF mutation. The complete mutational profile has been reported by Vergani et al. (25). Specifically, several genes found mutated in LM36/LM36R, including IGFR2, ARID1A, DDR2, MSH2, PRKDC and FGFR3, are wild-type in LM16/LM16R cells. Additionally, the mutational profile of CDKN2A and NRAS appears to be of interest. The former is found mutated only in LM16 and LM16R cells, whereas NRAS is mutated only in LM36R All the cell lines were cultured in RPMI-1640 medium (Lonza, Basel, Switzerland) supplemented with 10% FBS (Euroclone, Milan, Italy). The cells were cultured within 20 passages starting from thawing of frozen stock and routinely checked for mycoplasma contamination (Mycoalert, Lonza). Melanoma cells were verified for PLX4032 resistance and all the cells were authenticated by the Stem Elite ID System (Promega, Wisconsin, United States). PLX4032 (Selleckchem, Houston, TX, United States) was dissolved and diluted in dimethylsulfoxide (DMSO). Final DMSO concentration in medium never exceeded 0.25%. cDDP (Accord Healthcare Italia, Milan, Italy) was diluted in saline. Temozolomide (TMZ, Selleck Chemicals, Aurogene Srl, Rome, Italy) was primarily dissolved in DMSO and diluted in water. The KiSS1-derived peptide KP54 was obtained from Anaspec (DBA Italia, Milan, Italy). Exponentially growing cells were seeded in 12-well plate (5000 cells/mL) and, 24 h later, exposed for 72 h to different concentrations of drugs. At the end of the treatment, cells were detached and counted using coulter counter (ZB1, Coulter Electronics). The cellular sensitivity to the drugs is determined as a percentage of cell growth with respect to the untreated control. The IC_50_ is the drug concentration causing 50% reduction of cell growth. RI is the ratio between IC_50_ of resistant cell line and IC_50_ of the sensitive cell line.

### Quantitative real time polymerase chain reaction

Gene expression levels of KiSS1 and KiSS1R were analyzed by qRT-PCR according to standard methods in untreated cells. Twenty-four hours after seeding, cells were harvested and total RNA isolated using RNeasy Plus Mini kit (Qiagen, Hilden, Germany). The RNA was reverse transcribed by High Capacity cDNA Reverse Transcription kit (Thermo Fisher Scientific, Monza, Italy). The following TaqMan assays were used: Hs.PT.58.2731441 for KiSS1, Hs.PT.58.27127688 for KiSS1R, and Hs02758991_g1 for GAPDH (Thermo Fisher Scientific). Technical triplicate reactions were carried out with a 7900HT Fast Real-Time PCR System (Thermo Fisher Scientific) and data were acquired through the Sequence Detection Systems (SDS) 2.4 software. Reactions were in a 10 µL volume comprising cDNA (2.5 µL), master mix (5 µL, TaqMan Universal Fast PCR Master Mix, Thermo Fisher Scientific) and the specific assay (0.5 µL). The relative quantification (RQ) manager software (Thermo Fisher Scientific) was used to determine relative expression levels in resistant variants using parental cells as calibrator (23).

### Quantitative analysis of KiSS1 in melanoma cells and culture medium

KiSS1 levels expressed by the cells or released into the culture medium were measured by ELISA (Human Metastasis Suppressor KiSS-1 kit, Cusabio, Houston, TX, USA), according to the manufacturer’s instructions for quantitative analysis. Cells (26700 cells/cm^2^) were cultured for 24 h before the 24 h-treatment with PLX4032. At the end of treatment, cells and the corresponding culture media were recovered. Adherent cells were counted to allow normalization of the KiSS1 peptide levels. Cells were lysated and culture media clarified by centrifugation at 13,000 rpm for 5 min. Aliquots of cell extracts and culture media were used for the ELISA. A calibration curve was fitted by plotting the mean plate standard’s absorbance (dependent variable) as a function of the known KiSS1 concentrations of the standard (independent variable). This curve was then used to estimate the unknown starting concentration in the test samples. Three independent experiments were performed and the mean value ± standard deviation (SD) was calculated.

### KiSS1 silencing in melanoma cell lines

Seventy two hours after seeding in 75 cm^2^ flasks (6600 cells/cm^2^), LM16 and LM16R cells were transfected using Opti-MEM transfection medium (Gibco by Thermo Fisher Scientific, Waltham, MA, USA) and RNAiMax (Thermo Fisher Scientific), with 30 nM of small interfering RNA (siRNA) to KiSS1 (Silencer Select siRNA s194584, Thermo Fisher Scientific), and control siRNA (Silencer Select Negative Control #2 siRNA, Thermo Fisher Scientific). After 5h, the transfection was stopped by adding complete medium and 48 h later cells were harvested and seeded in 12-well plates (12000 cells/cm^2^). Cells were then treated with PLX4032 for 48 h. Knockdown efficiency was evaluated by qRT-PCR at the beginning and at the end of the drug treatment.

### Apoptosis analyses

The Annexin V-binding assay (Immunostep, Salamanca, Spain) was used to measure the apoptosis induction following drug exposure. Cells were treated for 48 h with PLX4032, cDDP, TMZ, KP54 alone or with their simultaneous combinations. After washing with cold phosphate-buffered saline (PBS), cells were processed according to manufacturer’s protocol. Annexin V-binding was examined by flow cytometry (BD Accuri, Becton Dickinson, Milan, Italy) by acquiring ten thousand events for each sample. Instrument software (Becton Dickinson) was used to analyze the results.

Apoptosis was also evaluated by measuring the activation of caspase 3/7 as well as caspase 8 by luminescent Caspase Glo 3/7 assay System or Caspase-Glo 8 Assay System (Promega, Fitchburg). Cells were seeded in 96-well plates (7,000 cells/well in 100 µL of medium) and 24 h later treated with PLX4032, cDDP, TMZ or KP54 alone or with their simultaneous combinations. After 48 h, the activation of caspases was determined according to the manufacturer’s instructions. Relative luminescence units (RLU) were normalized with respect to the total protein content of each well to correct for the growth inhibitory effect of the treatment. Protein content was assayed by the BCA method.

### Cell-cycle analysis

Twenty four hours after seeding in 75 cm^2^ flasks (10000 cells/cm^2^), LM16, LM36, LM16R and LM36R cells were exposed for 48 h to KP54, PLX4032 or to the simultaneous combination of KP54 and PLX4032. After treatment, cells were washed, fixed in ice-cold 70% ethanol, and stored at −20°C. After rehydration in PBS, cells were stained with 10 μg/mL propidium iodide (Sigma-Aldrich, St. Louis, MO, USA) in PBS containing RNase A (66 units/mL; Sigma-Aldrich) for 18 h. The samples were processed by flow cytometry (BD Accuri, Becton Dickinson) by acquiring 30 thousand events for each sample. Kaluza analysis software (2.1 version, Beckman Coulter) was used to analyze the results.

### Western blot analysis

Western blot analysis was carried out as previously described (23). Protein lysates fractionated by SDS-PAGE were blotted on nitrocellulose membranes. Blots were pre-blocked in PBS containing 5% (w/v) dried no fat milk and incubated overnight at 4° C with antibodies to anti p27^kip1^ (BD Biosciences, Franklin Lakes, NJ, USA) and anti-actin (Sigma-Aldrich). Blots were developed by chemo-luminescence (GE Healthcare, Chicago, IL, USA). Secondary antibodies were from GE Healthcare.

## Results

### Sensitivity of melanoma cell lines to PLX4032

In the present study, we used two pairs of vemurafenib (PLX4032)-sensitive and -resistant cells (LM16 and LM16R; LM36 and LM36R) to explore the possible interest of kisspeptins as modulator of response to vemurafenib in cells displaying sensitivity or resistance. Compared to LM16 and LM36, the resistant variants were 145.25 and 59.53 times more resistant to PLX4032, respectively (**Table 1**). Upon exposure to PLX4032, we observed changes in cell morphology suggesting the activation of cell death (**Supplementary Figure 1**).

**Table 1:** Sensitivity of melanoma cell lines to PLX4032^a^.

Cell lines	PLX4032 (IC_50_, µM)	RI
LM36	0.043 ± 0.01	/
LM36R	2.56 ± 0.55	59.53
LM16	0.08 ± 0.04	/
LM16R	11.62 ± 4.5	145.25

a Cell sensitivity was assessed by cell growth inhibition assay. Cells were seeded and 24 h later exposed to the drugs for 72 h. Cells were then counted using a cell counter. IC_50_ is defined as the drug concentration causing 50% reduction of cell growth. RI, Resistance Index; is the ratio between IC_50_ of resistant cell line and IC_50_ of the sensitive cell line. Experiments were performed in triplicate and data represent mean values ± SD.

### Analysis of the expression of KiSS1 and KiSS1 receptor and evaluation of kisspeptin levels in melanoma cell lines

**Figure 1:**
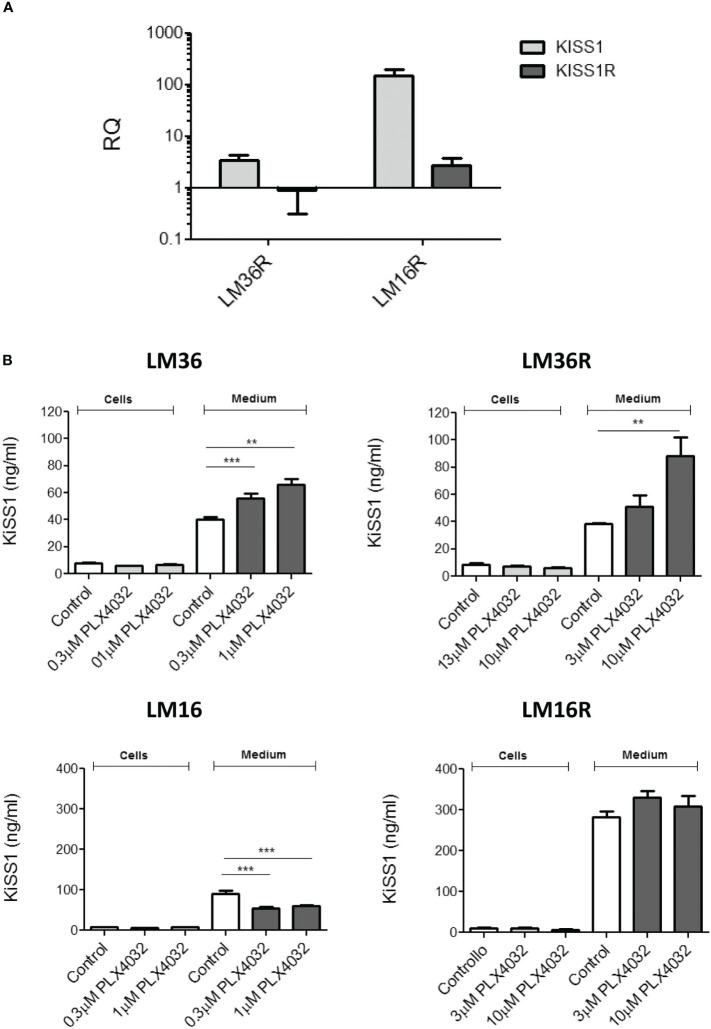
**(A)** Analysis of the expression of KiSS1 and KiSS1 receptor in melanoma cells. Twenty-four hours after seeding, exponentially growing cells were harvested and total RNA isolated. Gene expression levels of KiSS1 and KiSS1R were analyzed by qRT-PCR. Histograms represents the mean ± SD of 3 independent experiments. **(B)** Analysis of the levels of kisspeptins expressed or released by melanoma cells upon PLX4032 treatment. Twenty-four hours after seeding, cells were treated with PLX4032 for 24 h. Cells were then harvested and the levels of KiSS1 inside the cells or released into the medium measured by ELISA. ** p < 0.005; *** p < 0.0005 by one-way ANOVA followed by Bonferroni correction compared to single agents.

To characterize the cell models, the expression of KiSS1 and KiSS1R of melanoma cell lines was evaluated using qRT-PCR (**Figure 1A**). Specifically, compared to LM36R, LM16R show about 30-fold and 3-fold increased levels of KiSS1 and KiSS1R, respectively.

**Figure 2:**
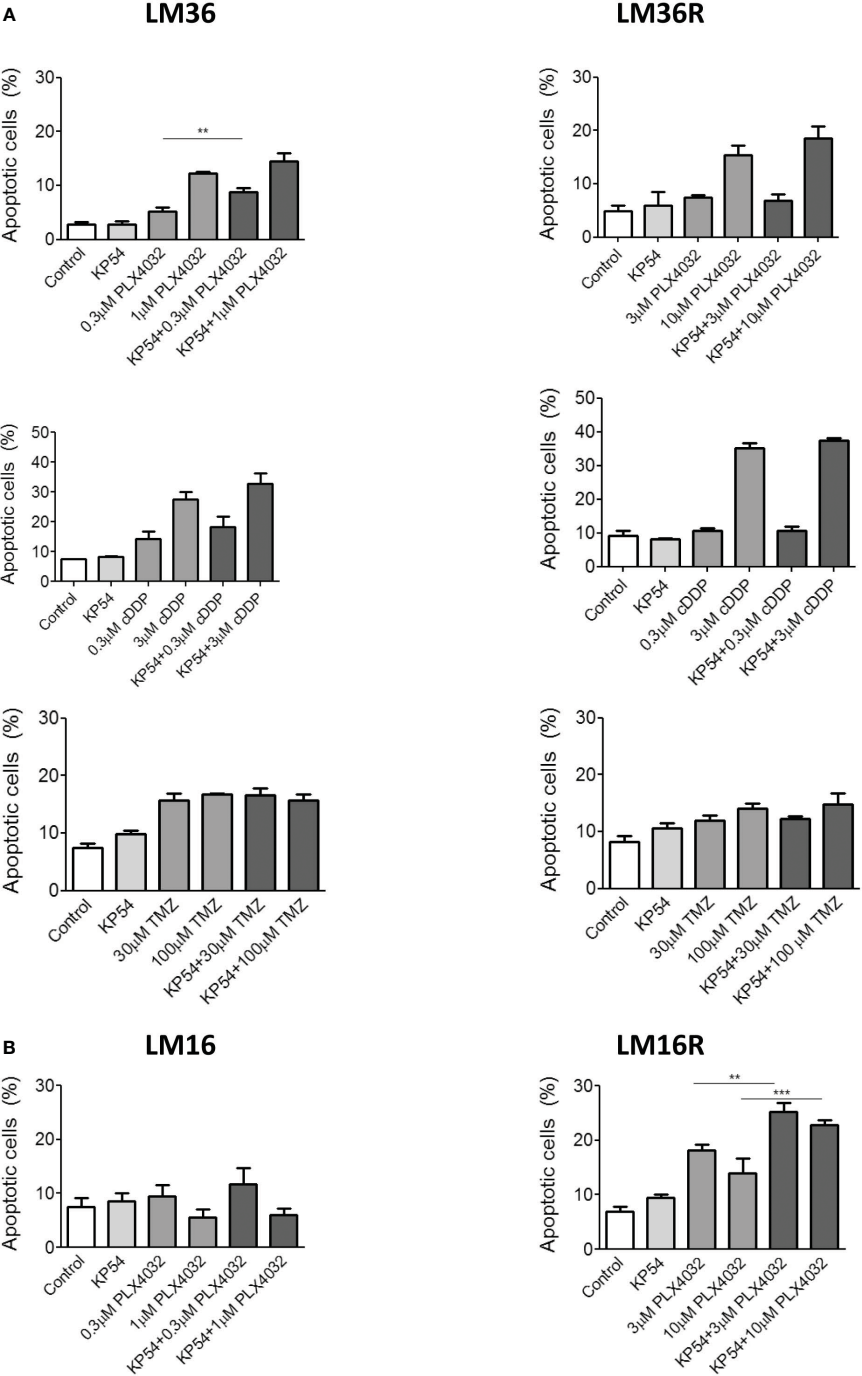
Apoptosis induced by the combination of PLX4032, cisplatin or temozolomide with KP54 in melanoma cells as assessed by Annexin V-binding assay. **(A)** Twenty four hours after seeding, LM36 and LM36R cells were exposed to PLX4032, cDDP or TMZ alone or to the combination with 500 ng/ml KP54 and harvested 48 h after treatment for analysis of apoptotic response. **(B)** Twenty four hours afrter seeding, LM16 and LM16R cells were exposed to PLX4032 alone or to the combination PLX4032 and 500 ng/ml KP54 and harvested 48 h after treatment for analysis of apoptotic response. Apoptosis quantitation was carreid out with the BD Accuri software. Histograms represent the mean ± SD of 3 independent experiments. ** p < 0.005; *** p < 0.0005 by one-way ANOVA followed by Bonferroni correction.

KiSS1 espression of melanoma cells and the levels of KiSS1 released into the culture medium were measured using ELISA (**Figure 1B**). Compared to LM16, LM16R cells show 3-fold increased KiSS1 levels released into the medium. Though no important differences in the released KiSS1 was observed for LM36R with respect to LM36, an appreciable and statistically significant secretion of KiSS1 was evidenced in both LM36 and LM36R cells, but not in LM16 and LM16R cells, upon treatment with PLX4032. KiSS1 expression was unaffected by the treatment with PLX4032 and was very similar among the cell lines considered. Of note, upon siRNA-mediated silencing of KiSS1, the sensitivity to PLX4032 of LM16 and LM16R cells resulted unaffected (**Supplementay Figure 2**).

### Analysis of apoptosis induction

Because we planned to use apoptosis as readout of the treatment efficacy, apoptosis induction was examined in response to treatment of melanoma cells with the BRAF inhibitor PLX4032 as well as to conventional antitumor agents such as cDDP and TMZ. Although cDDP is not used in melanoma therapy, the literature provides evidence of modulation of apoptosis by cDDP in head and neck and lung cancers (9, 10). Thus, cDDP treatment was included in our study. At first, apoptosis induction following PLX4032 exposure was examined in LM36 and LM36R melanoma cells. Moreover, LM36 and LM36R were exposed for 48 h to two different concentrations of PLX4032 alone or in combination with 500 ng/mL KP54 (10). A dose-dependent apoptosis induction was observed following the treatment of LM36 and LM36R with PLX4032 (**Figure 2**). No apoptosis was revealed upon KP54 exposure alone in both the cell lines. Compared to PLX4032 treatment, an increased number of apoptotic-positive cells was evidenced following the exposure to the combination PLX4032/KP54. This finding was statistically significant only in LM36, although a similar trend/behavior was evidenced in LM36R as well.

In addition, since KP54 combined with cDDP has been reported to result in a synergic interaction that potentiates the antitumor activity of cDDP in head and neck squamous cell carcinoma and lung cancer cells (9, 10), also the combination PLX4032/cDDP was considered. As expected, cDDP exposure resulted in a dose-dependent induction of apoptotis in LM36 and LM36R. Differently from what was observed following the treatment with the combination PLX4032/KP54, the exposure to cDDP/KP54 did not result in amelioration of the apoptosis induction with respect to cDDP as single agent. When using another DNA damaging agent of clinical interest in melanoma, i.e., TMZ, an appreciable induction of apoptosis was revealed in LM36 and LM36R cells. This behavior was independent of the drug concentration used and no implementation of apoptotic-positive cells was shown following the exposure to the combination TMZ/KP54 in sensitive and resistant cells. Of note, LM36 and LM36R showed similar sensitivity to cDDP (RI=1.43), while a collateral sensitivity to TMZ (RI=0.3) was observed for LM36R with respect to LM36 (**Supplementary Table 1**).

Besides, the analysis of apoptosis in LM16 and LM16R cells upon exposure to PLX4032 showed a modest apoptosis induction only in LM16R cells. Compared to untreated control, no induction of apoptosis was recognized in LM16 cells exposed to PLX4032, suggesting that the cells respond to treatment only inhibiting proliferation The combination PLX4032/KP54 did not improve the apoptotic-positive LM16 cells with respect to the exposure to PLX4032 alone. Conversely, KP54 significantly increased the PLX4032-induced apoptosis in LM16R cells.

**Figure 3:**
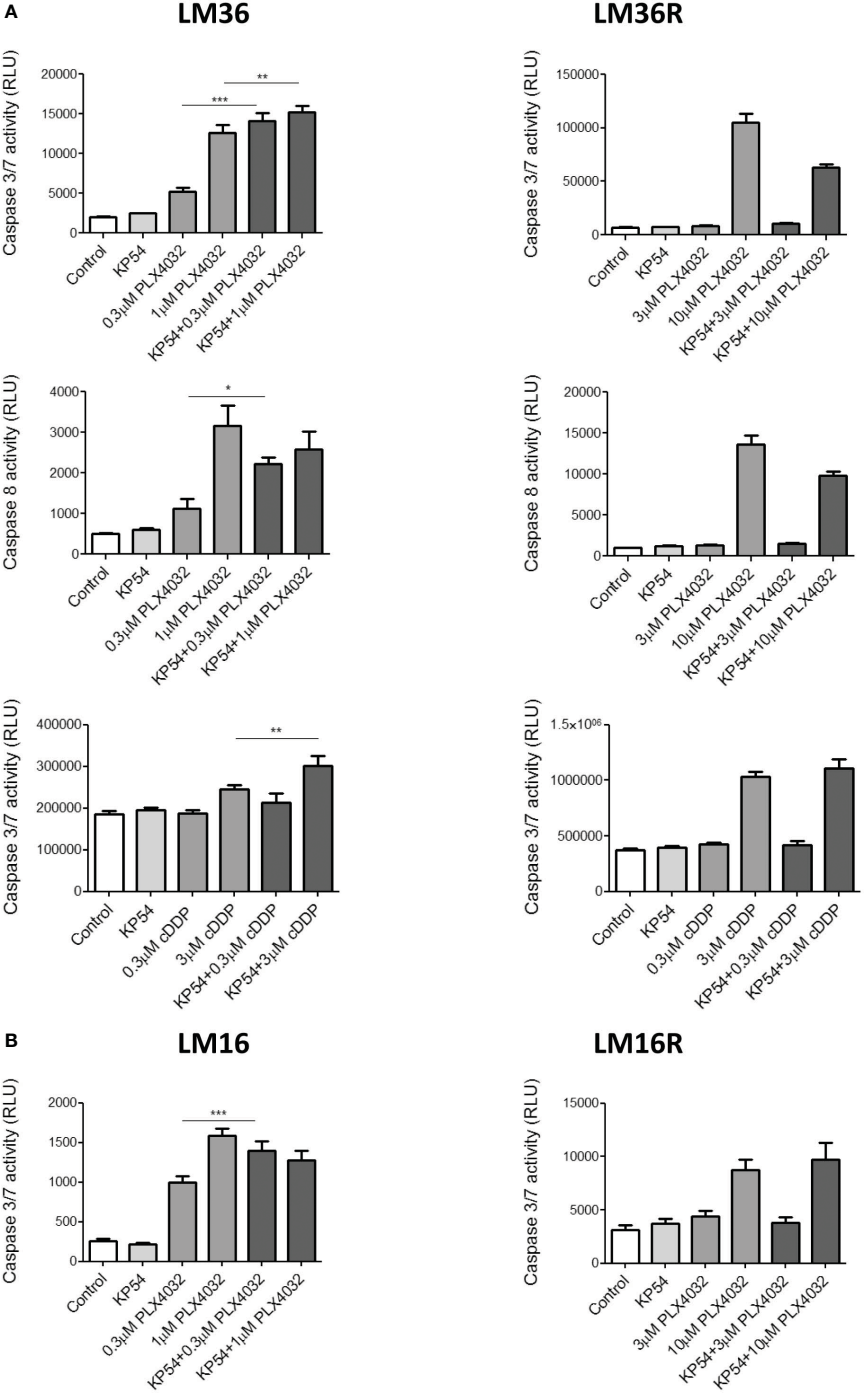
Caspase 3/7 and caspase 8 activation induced by the combination of PLX4032 or cisplatin with KP54 in melanoma cells. **(A)** Twenty four hours after seeding, LM36 and LM36R cells were exposed to PLX4032 or cDDP alone or to the combination with 500 ng/ml KP54 and harvested 48 h after treatment for the evaluation of caspase 3/7 or caspase 8 activation. **(B)** Twenty four hours after seeding, LM16 and LM16R cells were exposed to PLX4032 alone or to the combination PLX4032 and 500 ng/ml KP54 and harvested 48 h after treatment for the evaluation of caspase 3/7 activation. Histograms represent the mean ± SD of 3 independent experiments. * p < 0.05, ** p < 0.005; *** p < 0.0005 by one-way ANOVA followed by Bonferroni correction compared to single agents.

To better define the players of apoptosis induction, the activation of caspase 3/7 and 8 following drug exposure was evaluated (**Figure 3**). An increased activation of caspase 3/7 and 8 was evidenced in LM36 cells exposed to PLX4032. The combination of PLX4032 with KP54 implemented the activation of caspase 3/7 at both concentrations of PLX4032 considered and that of caspase 8 only at low concentration of PLX4032. The activation of caspase 3/7 and 8 was observed in LM36R exposed only to a high concentration of PLX4032. The exposure of LM36R cells to the combination of PLX4032 with KP54 resulted in reduced activation of both caspase 3/7 and 8. LM36 cells treated with cDDP slightly increased caspase 3/7 and this activation was significantly potentiated by the combination with KP54 only for the higher cDDP concentration. LM36R cells exposed to the higher cDDP concentration importantly increased the levels of activated caspase 3/7, which was not implemented upon the combination with KP54. No activation of caspase 3/7 was observed after the exposure to the lower concentration of cDDP alone or in combination with KP54. The treatment of LM16 cells with PLX4032 increased the activation of caspase 3/7. Following the exposure of LM16 cells to the combination of PLX4032 with KP54, the activation of caspase 3/7 was significantly implemented only for low concentration of PLX4032. The activation of caspase 3/7 was observed in LM16R cells only upon exposure to high concentration of PLX4032, and this activation was not implemented by the treatment with the combination PLX4032/KP54.

We also explored whether the advantage of the combination of KP54 and PLX4032 could be evident by examining cell viability by cell counting, thereby analyzing the combination efficacy in terms of proliferation inhibition. Compared to single drug exposure, the combination of KP54 with PLX4032, cDDP and TMZ did not impact on cell viability (**Supplementary Figure 3**). Additionally, since in our previous study we observed the induction of p27^kip1^ in melanoma cells treated with PLX4032 (24) and based on the observation that LM16 cells exposed to the combination of KP54/PLX4032 improve the PLX4032-mediated induction of p27^kip1^ (**Supplementary Figure 4**), we sought to investigate whether KP54 exposure perturbes the cell-cycle. With this approach, we found that a common feature of exposure to PLX4032 in LM16 cells was an accumulation in G1 (**Supplementary Table 2**), that was maintained with the combination KP54/PLX4032, a result supporting the occurrence of an antiproliferative response in these cells.

## Conclusions

The clinical management of metastatic melanoma has been revolutionized by the introduction of BRAF and MEK inhibitors. Despite the success achieved, persistent cures are still lacking and the development of drug resistance urgently requires the discovery of new drugs as well as innovative medical strategies. In this context, studies aimed at clarifying the molecular mechanisms subtending melanoma aggressiveness as well as response to treatment are expected to improve the medical management of this disease. In this study, we focused on the metastasis suppressor gene KiSS1, whose function as a modulator of apoptosis in head and neck and lung cancers has already been demonstrated (9, 10). Here, a role for KiSS1 as regulator of the cellular response to vemurafenib in melanoma cell lines all characterized by the BRAF V600E mutation has emerged. Pairs of melanoma cell lines sensitive and resistant to PLX4032, displaying different levels of KiSS1 and KiSS1R as well as a different pattern of KiSS1 release upon PL4032 exposure were considered to study whether the response to antitumor agents can be improved by the combination with KP54. Our results demonstrate that in melanoma models, the apoptotic response to the treatment with PLX4032 is improved following the combination with KP54. A contribution of KiSS1 released upon PLX4032 exposure to such an improvement is also likely, dependent on the fact that increased release is found in the cell lines in which apoptosis is increased by KP54 combination (LM36, LM36R, LM16R), but not in the LM16 cell line. The amelioration of the apoptotic process is evident both in LM16R and LM36 cell lines, with a statistically significant improvement of the percent of apoptotic cells upon treatment with the combination versus PLX4032 alone. Of note, the activation of caspase is observed in the parental cell lines (LM16 and LM36). In LM16 cells, a trend toward an increase of apoptotic cells is evident, thereby the discrepancy between quantitative analysis of apoptosis and caspase activation is only apparent. In LM16 cells, the lack of induction of apoptosis by PLX4032 *per se* suggests that this cell line responds to treatment with an antiproliferative effect and not with apoptosis induction. In LM36 cells, the activation of both caspase 3-7 and caspase 8 is observed and this implies that the process of apoptosis induction appears to involve both the intrinsic and the extrinsic apoptotic pathways. Since this behavior is observed also upon the combination of KP54 with cDDP, the study envisions a general mechanism of action of kisspeptins, supporting that the drug combination can potentiate the activity of antitumor drugs whose mechanism of action involves the induction of apoptosis. Importantly, the siRNA-mediated silencing of KiSS1 has negligible effects on cell growth. However, a reduced cell growth was observed in KiSS1-silenced cells exposed to 10 µM PLX4032. Although there might be a trend, according to the statistical analysis this reduction was not significant. Besides, no increase of apoptosis was observed. Thus, overall, it seems that the most promising approach to modulate response to vemurafenib is a gain of function approach based on the use of kisspeptins. The loss of function approach seems not to allow to easily dissect the contribution of KiSS1 to drug response likely becasue the phenomenon is multifactorial and compensatory signals could occur, thereby masking the phenotype of interest to us. Differently, the gain of function approach based on the use of KP54 triggers stronger cellular changes in terms of cellular response to treatment. Such a response may be variable in different cell lines, also depending on the specific molecular background. Indeed, the mutational profiles of the cell lines indicate a wide heterogeneity between parental cell lines and upon resistance acquisition. Of note, cell response seems to be the result of a balance among multiple factors, given that survival proteins (e.g., Bcl-2) may also be lost in resistant cells (data not shown). The research requires further studies to elucidate the molecular determinants implicated in the drug combination. In particular, deeper investigations aimed at clarifying the role played by the expression of KiSS1 and KiSS1R as well as the contribution of secreted KiSS1 by the tumor are needed.

In conclusion, our results indicate that, beyond its role as a metastasis suppressor, KiSS1 is critical in modulating the apoptotic response of melanoma to antitumor drug treatment and allows to the speculation that the combination with kisspeptins may improve response to chemotherapy treatment with vemurafenib.

## Data availability statement

The original contributions presented in the study are included in the article, further inquiries can be directed to the corresponding author.

## Ethics statement

Ethical approval was not required because the cell lines were developed in our institution in the context of previous projects approved by the ethical committee. The studies were conducted in accordance with the local legislation and institutional requirements.

## Author contributions

CG, CC, EC, LM and EV performed the experiments and data collection. GB, CG and PP wrote the first draft of the manuscript. GB, MR and PP contributed to the conception and design of the study and manuscript drafting. PP contributed to funding acquisition. All authors have provided contribution to data analysis, editing, and final approval of the manuscript.
