# Supplementary material for: Kisspeptin-mediated improvement of sensitivity to BRAF inhibitors in vemurafenib-resistant melanoma cells
Source: Front Oncol. 2023 Sep 18;13:1182853. doi: 10.3389/fonc.2023.1182853 (PMC10544897; doi:10.3389/fonc.2023.1182853)
Supplement: Supplementary file 1 [file DataSheet_1.pdf]

Supplementary Material

Kisspeptin-mediated improvement of sensitivity to BRAF inhibitors in vemurafenib-resistant melanoma cells

Carlotta Guzzetti [1†], Cristina Corno[1†], Elisabetta Vergani[2], Luca Mirra[1], Emilio Ciusani[3], Monica Rodolfo[2], Paola Perego[1]*, Giovanni L. Beretta[1]

[1]Molecular Pharmacology Unit, Department of Experimental Oncology, Fondazione IRCCS Istituto Nazionale dei Tumori Milan, Milan, Italy

[2]Unit of Immunotherapy of Human Tumors, Department of Experimental Oncology, Fondazione IRCCS Istituto Nazionale dei Tumori Milan, Milan, Italy

[3]Laboratory of Clinical Pathology and Medical Genetics, Istituto Neurologico Fondazione C. Besta, Milan, Italy

*** Correspondence:** Paola Perego paola.perego@istitutotumori.mi.it

[†]These authors have contributed equally to this work

Supplementary Table 1. Sensitivity of LM36 and LM36R melanoma cell lines to cisplatin and temozolomide[a]

Cell lines	cDDP (IC_{50}, µM)	RI	TMZ (IC_{50}, µM)	RI
**LM36**	0.51 ± 0.08	/	62.8 ± 1.75	/
**LM36R**	0.73 ± 0.03	1.43	19 ± 4.8	0.3

[a]Cell sensitivity was assessed by cell growth inhibition assay. Cells were seeded and 24 h later exposed to the drugs for 72 h. Cells were then counted using a cell counter. IC_{50} is defined as the drug concentration causing 50% reduction of cell growth. RI: Resistance Index, is the ratio between IC_{50} of resistant cell line and IC_{50} of the sensitive cell line. Experiments were performed in triplicate and data represent mean values ± SD.

Supplementary Table 2. Analysis of the cell cycle perturbations of melanoma cells exposed to PLX4032 and KP54[a]

LM36				LM36R			
**Drug**	**G1 (%)**	**S (%)**	**G2 (%)**	**Drug**	**G1 (%)**	**S (%)**	**G2 (%)**
**Control**	65.36±2.99	27.27±1.34	7.36±2.94	**Control**	68.58±1.84	16.64±1.99	14.77±3.84
**KP54**	66.74±3.88	23.75±2.96	9.5±0.93	**KP54**	68.02±3.59	22.59±4.63	9.38±1.99
**PLX4032 0.3µM**	83.76±2.75	11.29±2.55	4.94±0.57	**PLX4032 3µM**	73.51±1.18	18.07±1.13	8.42±0.509
**PLX4032 1µM**	87.37±5.46	7.52±4.34	4.71±1.26	**PLX4032 10µM**	85.03±2.46	7.38±1.95	7.56±2.31
**KP54/ PLX4032 0.3µM**	86.40±2.07	9.27±1.60	4.32±0.86	**KP54/ PLX4032 3µM**	73.65±1.15	18.29±3.96	8.05±2.98
**KP54/ PLX4032 1µM**	87.38±3.18	8.49±2.83	3.94±1.34	**KP54/ PLX4032 10µM**	85.95±0.745	9.13±2.47	4.91±1.73

LM16				LM16R			
**Drug**	**G1 (%)**	**S (%)**	**G2 (%)**	**Drug**	**G1 (%)**	**S (%)**	**G2 (%)**
**Control**	53.96±1.31	35.06±0.78	10.97±1.56	**Control**	59.73±3.36	27.35±4.71	12.91±1.38
**KP54**	56.71±1.06	29.66±1.67	13.62±2.61	**KP54**	51.75±5.17	35.46±5.15	12.77±1.72
**PLX4032 0.3µM**	90.07±0.92	6.83±0.32	3.09±0.62	**PLX4032 3µM**	65.45±3.66	26.14±3.72	8.3±1.18
**PLX4032 1µM**	89.78±1.25	3.97±1.40	6.23±1.76	**PLX4032 10µM**	73.65±3.89	19.48±3.65	6.86±0.48

**KP54/ PLX4032 0.3µM**	87.06±1.50	8.40±1.09	4.53±0.73	**KP54/ PLX4032 3µM**	63.75±4.25	25.31±5.07	10.92±1.30
**KP54/ PLX4032 1µM**	90.20±2.71	5.77±2.68	4.01±0.50	**KP54/ PLX4032 10µM**	79.04±1.82	13.62±0.06	7.34±1.76

[a]Twenty-four hours after seeding, cells were exposed to PLX4032 and KP54 alone or in combination for 48 h. Cells were then harvested and processed for the analysis by flow cytometry. Experiments were performed in triplicate and data represent mean values ± SD.

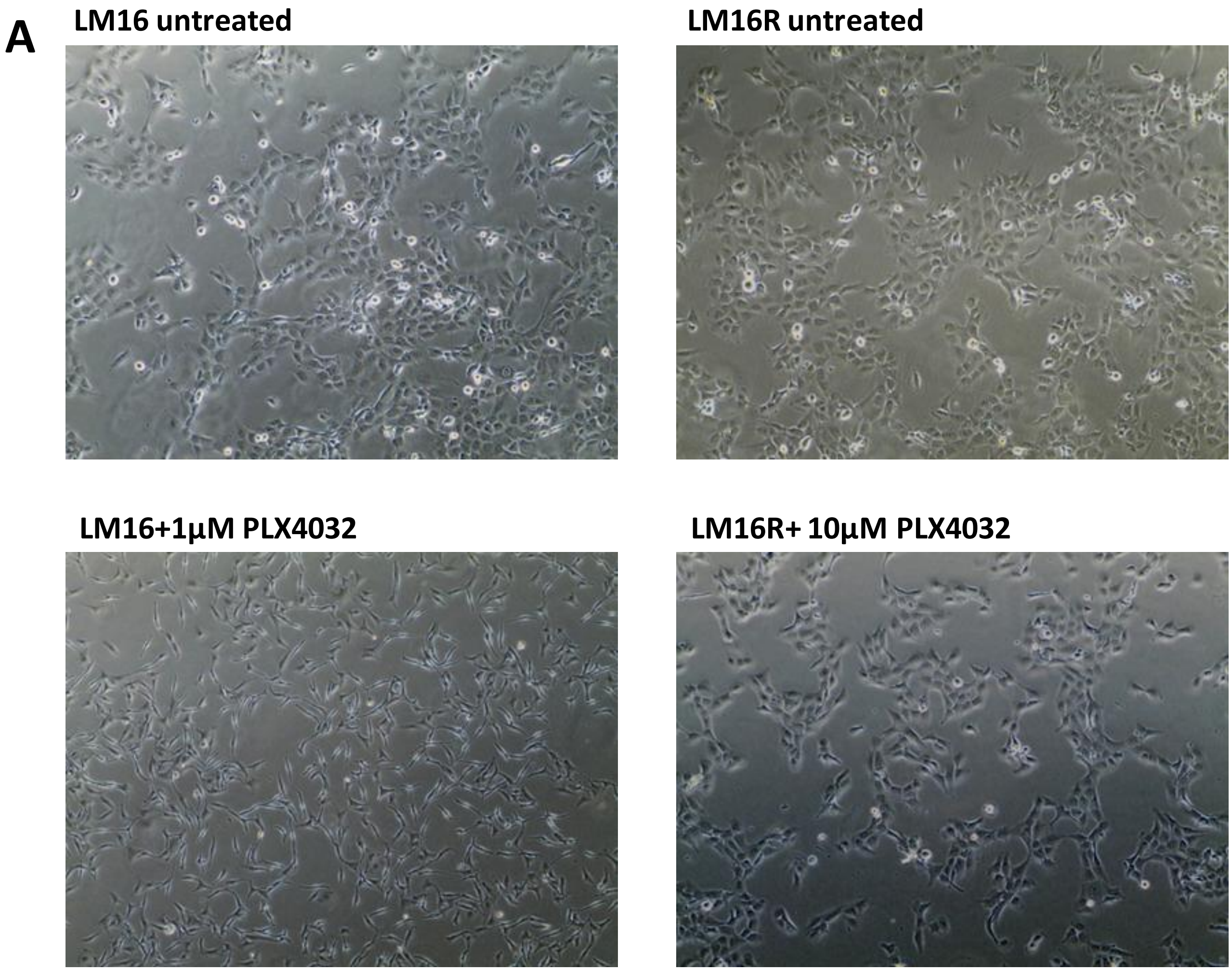
A
LM16 untreated
LM16R untreated
LM16+1µM PLX4032
LM16R+ 10µM PLX4032

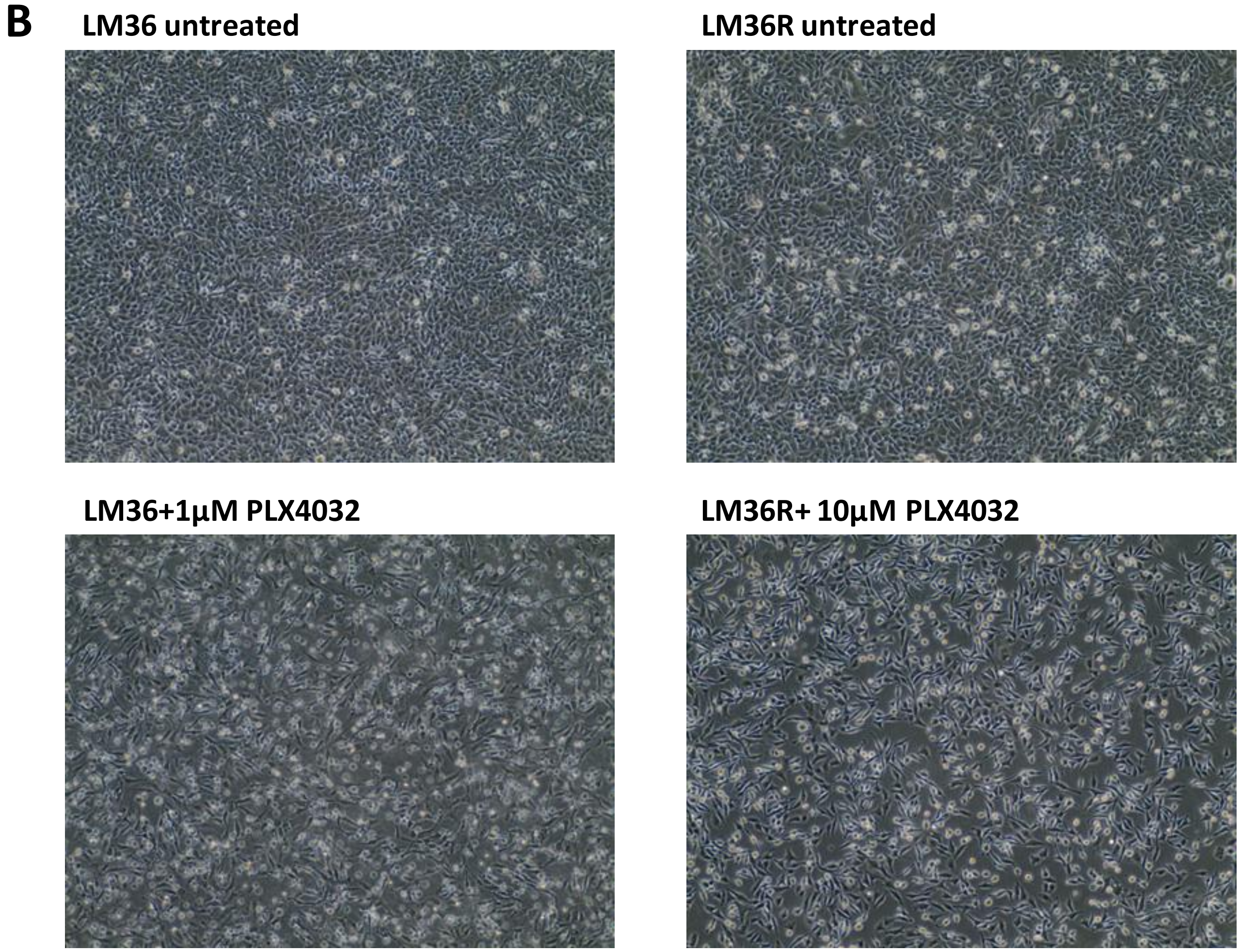

Supplementary Figure 1. Morphology of melanoma cells upon exposure to PLX4032. Following 48 h exposure to PLX4032, cells were observed under the microscope (EVOS XL Core Imaging System, Thermo Fisher Scientific, Monza, Italy) and the images acquired. LM16 and LM16R cells are shown in A. LM36 and LM36R are shown in B. Slides were viewed using an EVOS XL core microscope with a 10x objective. Images were taken using the built in EVOS XL core camera and acquisition software changing to grey scale.

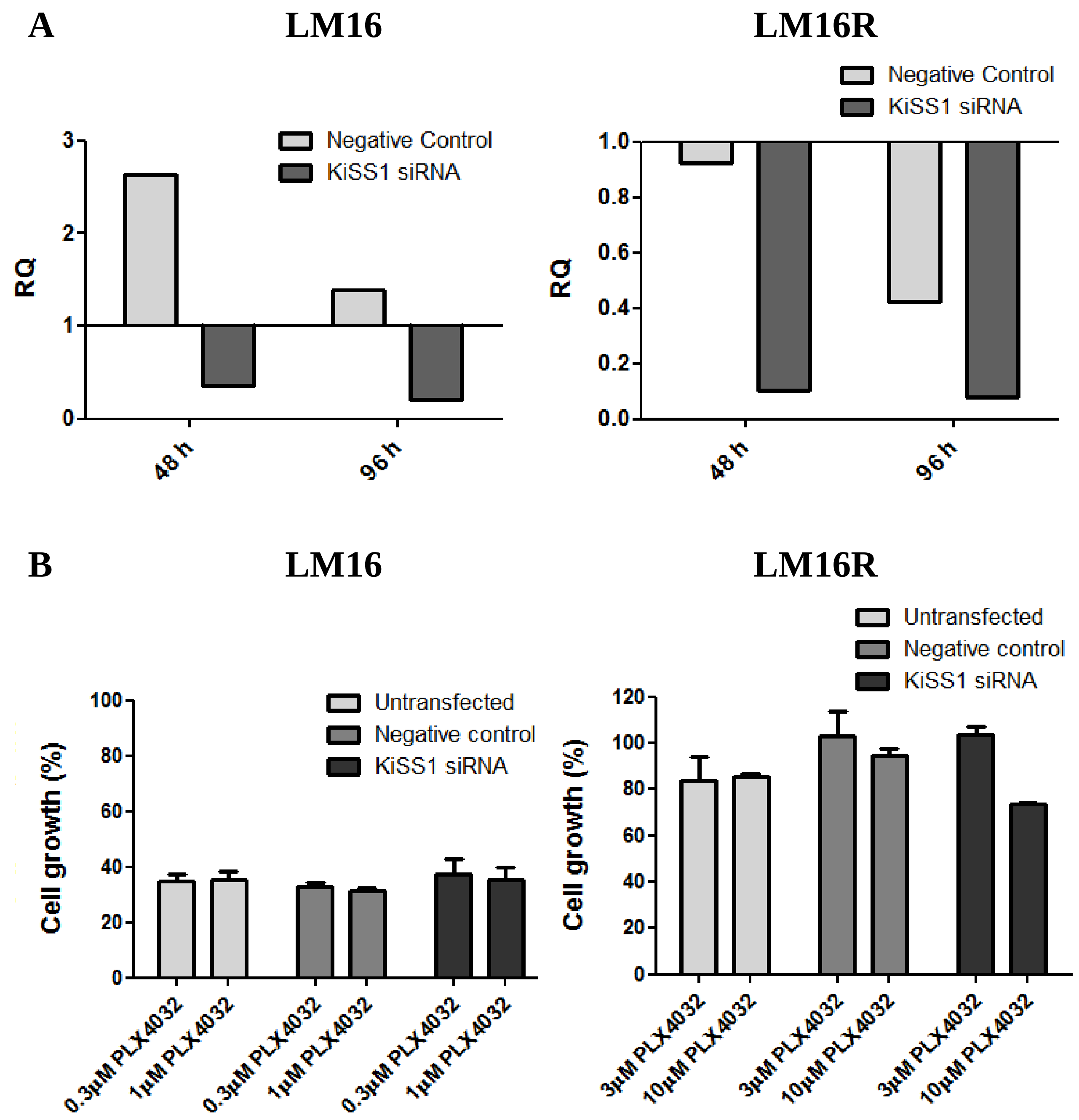

Supplementary Figure 2. KiSS1 silencing and evaluation of PLX4032 sensitivity in LM16 and LM16R cells. A) Seventy-two hours after seeding, exponentially growing cells were silenced for 48 h with KiSS1-directed siRNA. Cells were then harvested and total RNA isolated. Gene expression levels of KiSS1 were analyzed by qRT-PCR. B) Analysis of the sensitivity to PLX4032 upon KiSS1 silencing. Cell sensitivity was assessed by cell growth inhibition assay. KiSS1-silenced cells were exposed to PLX4032 for 48 h. Cells were then counted using a cell counter. The levels of KiSS1 at the end of the treatment (96h from the beginning of the silencing) were measured by qRT-PCR. Experiments were performed in triplicate and data represent mean values ± SD.

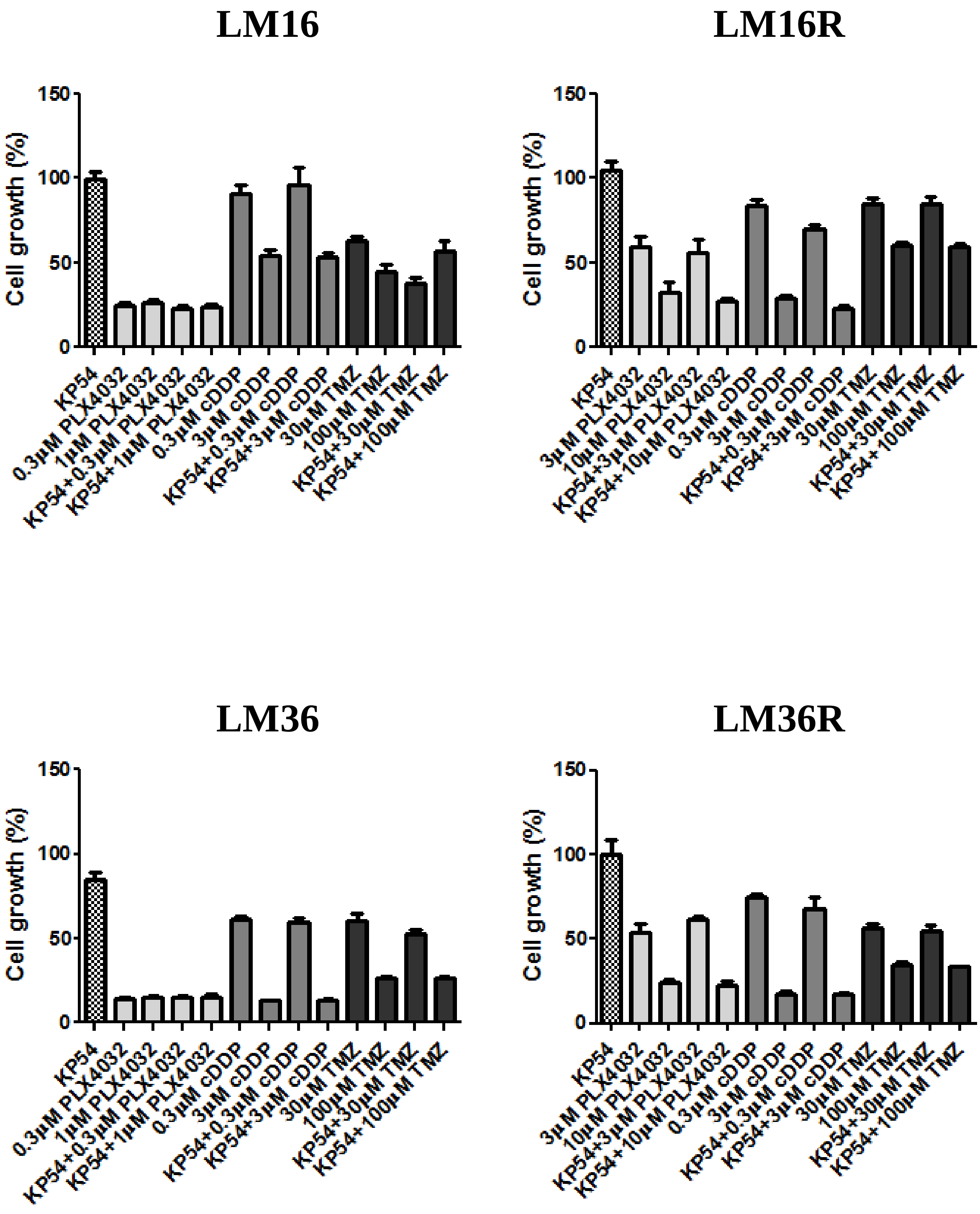

Supplementary Figure 3. Effect of the combination of KP54 with vemurafenib, cisplatin and temozolomide on the viability of melanoma cell lines. Cell sensitivity was assessed by cell growth inhibition assay. Twenty-four hours after seeding, cells were exposed to the drugs alone or to the simultaneous combination with KP54 for 72 h. At the end of the treatment, cells were harvested and counted using a cell counter. Experiments were performed in triplicate and data represent mean values ± SD.

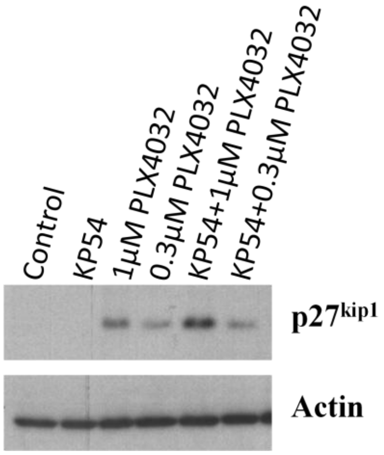

Supplementary Figure 4. The combination of KP54 and vemurafenib increases the expression of p27^{kip1}. Twenty-four hours after seeding, LM16 ells were exposed to the drugs alone or in simultaneous combination for 48 h. Cells were then harvested for protein extraction. Samples were loaded on SDS-PAGE. Actin represents control for loading.
